# High-coverage methylation data of a gene model before and after DNA damage and homologous repair

**DOI:** 10.1038/sdata.2017.43

**Published:** 2017-04-11

**Authors:** Antonio Pezone, Giusi Russo, Alfonso Tramontano, Ermanno Florio, Giovanni Scala, Rosaria Landi, Candida Zuchegna, Antonella Romano, Lorenzo Chiariotti, Mark T. Muller, Max E. Gottesman, Antonio Porcellini, Enrico V. Avvedimento

**Affiliations:** 1 Dipartimento di Medicina Molecolare e Biotecnologie Mediche, Istituto di Endocrinologia ed Oncologia Sperimentale del C.N.R., Università Federico II, Napoli 80131, Italy; 2 Dipartimento Biologia, Università Federico II, Napoli 80131, Italy; 3 Epigenetics Division, TopoGEN, Inc., 27960 CR319, Buena Vista, Colorado 81211, USA; 4 Institute of Cancer Research, Columbia University Medical Center, New York, New York 10032, USA

**Keywords:** DNA methylation, Bioinformatics, Data processing

## Abstract

Genome-wide methylation analysis is limited by its low coverage and the inability to detect single variants below 10%. Quantitative analysis provides accurate information on the extent of methylation of single CpG dinucleotide, but it does not measure the actual polymorphism of the methylation profiles of single molecules. To understand the polymorphism of DNA methylation and to decode the methylation signatures before and after DNA damage and repair, we have deep sequenced in bisulfite-treated DNA a reporter gene undergoing site-specific DNA damage and homologous repair. In this paper, we provide information on the data generation, the rationale for the experiments and the type of assays used, such as cytofluorimetry and immunoblot data derived during a previous work published in Scientific Reports, describing the methylation and expression changes of a model gene (GFP) before and after formation of a double-strand break and repair by homologous-recombination or non-homologous-end-joining. These data provide: 1) a reference for the analysis of methylation polymorphism at selected loci in complex cell populations; 2) a platform and the tools to compare transcription and methylation profiles.

## Backdground & Summary

Only recently has the extreme degree of polymorphism of DNA methylation become increasingly appreciated with the higher coverage and evolution of next-generation sequencing (NGS) technology.

We and others have provided evidence that: 1. somatic methylation is induced by DNA damage and homologous repair^1,2^; 2. methylation is highly polymorphic^3,4^; 3. the methylation pattern is initially unstable after repair and eventually stabilizes^3–5^. In a recent paper, published in *Scientific Reports*^6^, we demonstrated that DNA damage and repair generates clones with distinct GFP gene expression levels, which are marked by different DNA methylation patterns^6^. Bisulfite sequencing is the gold standard of DNA methylation analysis. It uses the direct sequencing of chemically-treated DNA to identify methylated cytosines at the single-nucleotide level. Genome- wide sequencing of bisulfite-DNA is unbiased with regard to the sequence representation, but is limited in the coverage of a single locus. In addition, the sequenced molecules represent a statistical collection of methylated cytosines derived from physically different molecules. Thus, epi-haplotypes or epi-polymorphisms linking *in cis* several CpGs in the same DNA molecule cannot be deduced from these sequences. For this reason, we have employed a reductionist approach to study induction and variation of somatic DNA methylation. In particular, we have sequenced DNA molecules with the same 5′ and 3′ ends. Each methylated sequence represents an individual allele differing only in the methylation of specific CpGs (epiallele). The number of different individual epialleles in the population provides the information on the frequency and the degree of epi-polymorphism.

**Figure 1:**
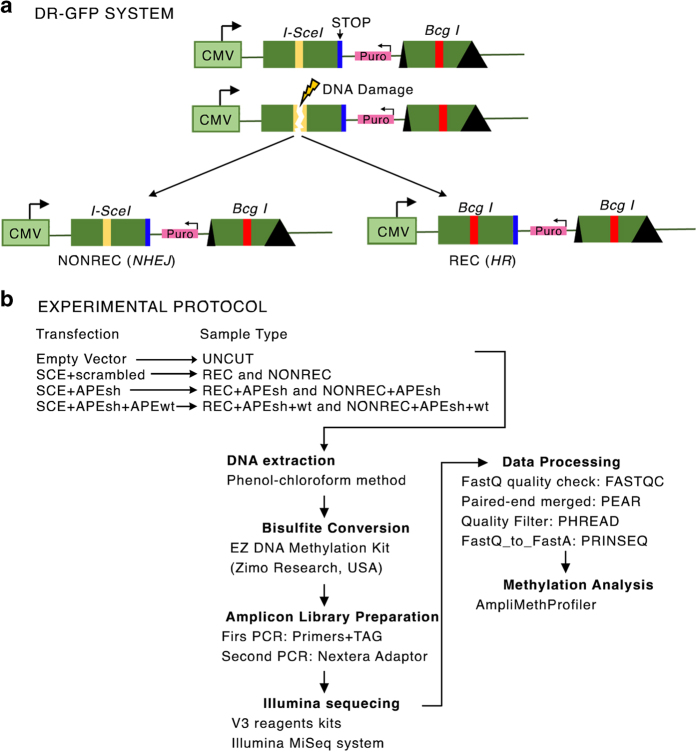
DRGFP system and the experimental protocol. (**a**) Schematic diagram of the DRGFP system. *I-SceI* (yellow line) indicates the site cleaved by the meganuclease I-SceI. The *I-SceI* site is converted by HR into a new site for the enzyme BcgI (*Bcg I* red line). The first and the second cassettes are shown. Cassette II is not transcribed. (**b**) Schematic representation of the experimental protocol. HeLa DRGFP cells were transfected with (1) SCE+scrambledshRNA; (2) SCE+APEsh; (3) SCE+APEsh+APEwt (on the left). Recombinant (REC) and non-recombinant or NHEJ (NONREC) molecules were purified following each transfection. UNCUT represents control plasmid-transfected HeLa DRGFP cells. The arrows indicate the steps and the procedures undertaken in analyzing the DNA methylation data.

**Figure 2:**
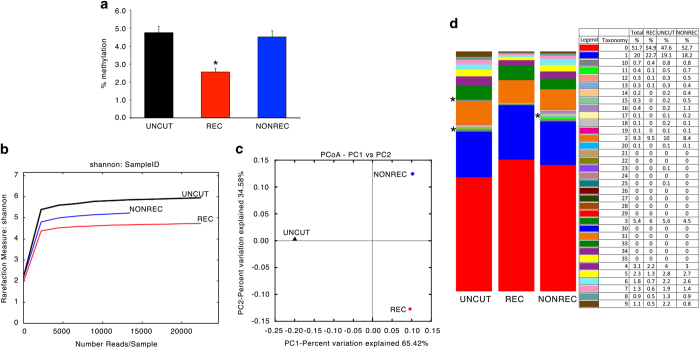
Qualitative DNA methylation profiles in recombinant and non-recombinant GFP molecules. (**a**) Quantitative methylation analysis of GFP molecules derived from the samples indicated in Fig. 1. In all, 41 CpGs are present in the fragment analysed and are located in the cassette I. The sequence of the cassette I in control or NONREC molecules has been transformed into the recombinant version to compare identical primary sequences. Data were expressed as the mean±s.e.m. (*n*=41); *P<0.01 (paired *t-*test) comparing REC versus UNCUT or versus NONREC. (**b**) Shannon diversity index between REC (red) and NONREC (blue) and control UNCUT (black). (**c**) Principal component analysis of REC (red) and NONREC (blue) and control UNCUT (black). (**d**) Methylation content (taxonomy) of REC and NONREC and control UNCUT.

The methods we have used are an expanded version of the descriptions published in Scientific Reports^6^ to analyze DNA methylation polymorphism in a GFP^+^ population after DNA damage and repair (ref. 6, Data Citation 1). Briefly, engineered HeLa cells were transfected with a vector expressing I-SceI (SCE) generating a single double strand break (DSB)/genome in one GFP copy (cassette I) which can be repaired from the second copy (cassette II) by homologous recombination (HR), yielding GFP^+^ clones (Fig. 1). In all, 75–90% of the cells are repaired by non-homologous end-joining (NHEJ) with or without small deletions at the *I-SceI* site^7^. Importantly, GFP^+^ cells can arise in this system only by HR^1,7^. The data indicate that extent of methylation does not distinguish between uncut control (SAMD00063102, SAMD00063106, Data Citation 1) versus non-recombinant or recombinant DNA molecules (SAMD00063103, SAMD00063107, Data Citation 1) (Fig. 2a), whereas the number of methylated species (rarefaction index) in each sample shows recombinant molecules more similar to non-recombinant or NHEJ molecules than to undamaged control cells (variation 34 versus 65%) (Fig. 2b,c). However, the various samples may contain the same number of species but with a different composition. The species composition in the various samples shows that some families present in control and non-recombinant cells are absent from the recombinant clones (indicated with *). The species composition of uncut control and non-recombinant molecules is very similar (Fig. 2d).

This system can be used to determine whether the methylation profiles (epi-haplotypes) of GFP molecules before or after recombination differ and permanently mark specific repair events or whether they drift over time.

## Methods

This section includes and extends the information present in the original manuscript^6^.

### Cell culture, transfections and plasmids

HeLa cells lines were cultured at 37 °C in 5% CO_2_ in RPMI medium supplemented with 10% foetal bovine serum (Invitrogen, MA, USA), 1% penicillin-streptomycin, and 2 mM glutamine. HeLa-DR-GFP cells were obtained by stable transfection of HeLa cells with the pDR-GFP plasmid (Fig. 1) as described in ref. 1. The plasmids encoding shRNA-APE1 were constructed with the following oligonucleotides: sense, 5′- GATCCCCCCTGCCACACTCAAGATCTGCTTCAAGAGAGCAGATCTTGAGTGTGGCAGGTTTTTGGAAA-3′; and antisense, 5′-AG CTTTTCCAAAAACCTGCCACACTCAAGATCTGCTCTCTTGAAGCAG ATCTTGAGTGTGGCAGGGGG-3′. These sequences were drawn as described in ref. 8 and were designed to recognize and bind to a 21-base sequence (underlined) placed 175 nucleotides downstream of the AUG initiation codon of the human APE1 gene. As a control, we used the following scrambled oligonucleotide sequences: sense, 5′- GATCCCCAGTCTAACTCGCCACCCCGTATTCAAGAGATACGGGGTGGCGAGTTAGACTTTTTTGGAAA-3′; antisense, 5′- AGCTTTTCCAAAAAAGTCTAACTCGCCACCCCGTATCTCTTGAATACGGGGTGGCGAGTTAGACTGGG-3′. These sequences do not pair with any human cDNA (http://www.ncbi.nlm.nih.gov/blast/). The sequences were cloned into Bgl II and Hind III restriction sites of pSUPER vector (Oligoengine) to form the so-called pSUPER- APE1 vector. APE1 silencing was measured by western blot (see Technical Validation or Data Citation 2). Additional information is provided in ref. 6.

### DNA extraction

Genomic DNA extraction was performed using the following protocol: a the cellular pellet was resuspended in 10 mM TRIS (pH 7.8) and 50 mM NaCl solution (2×10^7^ cells per ml). After the addition of 1% SDS the sample was gently mixed. Proteinase K, at a final concentration of 200 μg ml^−1^, was added and the mixture was incubated at 55 °C overnight. The following day, hot NaCl solution (70 °C) was added to the mixture at a final concentration of 1.5 M and the DNA was extracted with chloroform. The DNA was ethanol precipitated, dried, and resuspended in TE buffer.

### FACS analysis

HeLa-DR-GFP cells were harvested and re-suspended in 500 μl of PBS at a density of 10^6^ cells per ml. Cell viability was assessed using propidium Iodide (PI) staining: before FACS analysis cells were incubated with 3 μM PI for 10 min. Cytofluorimetric analysis was performed using a 9,600 Cyan System (Dako Cytometrix) and PI positive cells were excluded from the analysis by gating the PI-negative cells on a FSC-Linear versus FL2H-Log plot; GFP+ cells were identified with a gate (R1) on a FL1H-Log versus SS-Log plot. Rec L and Rec H cells were identified through a FL1H histogram of the R1-gated cells with 2 range-gate (see Technical Validation and Data Citation 3). The same gate was used for all flow cytometry experiments.

Comparison of the population was performed using the FlowJo software (Chi-Squared Test). Differences in fluorescence intensity (mean) were determined using the matched pairs Student’s *t* test.

### Bisulfite treatment and amplicon library preparation

2 μg of genomic DNA were converted with ‘C/T conversion reagent’ employing the EZ DNA Methylation Kit (Zymo Research, USA) and eluted in 50 μl of H_2_O following the manufacturer’s instruction. We generated an amplicon library of bisulfite-treated DNA using a double step PCR strategy. In the first PCR reaction, we amplified fragments ranging in size between 500–550 bp (all primers pairs are reported in Table 1). The 5′ ends of these primers contain overhang adapter sequences (Fw: 5′- TCGTCGGCAGCGTCAGATGTGTATAAGAGACAG-3′, RV: 5′- GTCTCGTGGGCTCGGAGATGTGTATAAGAGACAG-3′) that will be used in the second step to add multiplexing indices and illumina sequencing adapters. First PCR was performed using the ‘FastStart High Fidelity PCR System’(Roche) under the following thermo cycle conditions: one cycle at 95 °C for 2 min followed by 30 cycles at 95 °C for 30 s, at TM 50 °C for 45 s, at 72 °C for 60 s, followed by a final extension step at 72 °C for 10 min. Reactions were performed in 20 μl total volumes: 2 μl 10× reaction buffer, 1 μl of 10 mM dNTP mix, 1 μl of 4 μM forward and reverse primers, 3.6 μl MgCl_2_ 25 mM, 2–4 μl bisulfite template DNA, 0.25 μl FastStart Taq, and H_2_O up to a final volume of 30 μl. Five μl of first PCRs were used to check product size on 1.5% agarose gel. To eliminate small DNA fragments (primers dimers), we used 20 μl of AMPure purification magnetic beads (Beckman-Coulter, Brea, CA, USA) following the manufacturer’s protocol. Second PCR was performed in 50 μl: 5 μl 10× reaction buffer, 2.5 μl of 10 mM dNTP mix, 5 μl forward and reverse ‘Nextera XT’ primers (Illumina, SanDiego, CA, USA), 6 μl MgCl_2_ 25 mM, 5 μl of first PCR product, 0.4 μl FastStart Taq, and H_2_O up to a final volume of 50 μl. Thermo-cycle settings were: one cycle at 95 °C for 2 min followed by 8 cycles at 95 °C 30 s, 55 °C for 40 s, 72 °C for 40 s, followed by a final extension step at 72 °C for 10 min. Another purification step was performed with 50.8 μl of AMPure beads and all amplicons were quantified using the Qubit 2.0 Fluorometer. The quality of each amplicon was checked with the Agilent 2,100 Bioanalyzer using the DNA 1,000 Kit (Agilent Technologies, Santa Clara, CA, USA) according to the manufacturer’s instructions. Amplicons were pooled at equimolar ratio and then diluted to final concentration of 8 pM. 15% (v/v) of Phix control libraries (Illumina, San Diego, CA, USA) was combined with normalized library to increase diversity of base calling during sequencing. The amplicons library was subjected to sequencing using V3 reagents kits in the Illumina MiSeq system (Illumina, San Diego, CA, USA). Pair-end sequencing was carried out in 281 cycles per read (281×2). An average of 25,000 reads were used for further analysis.

## Data Records

All data records are available to be downloaded from Figshare, in which Data Records 1 were deposited and released with the original publication by Russo *et al.*^6^.

### Data records 1

The fastq files obtained from the DNA-seq libraries were deposited in the DDBJ database under the DRA Accession Number (Data Citation 1). The processing of all fastq samples is summarized in Fig. 1. The list of fastq is summarized in Table 2.

### Data records 2

The western blot files were deposited in the Figshare dataset under Digital Object Identifier (Data Citation 2).

### Data records 3

The fcs files obtained from flow cytometric analysis were deposited in the Flow Repository database under ID (Data Citation 3).

## Technical Validation

These data are the results of two technical replicates of the independent sequencing of (+) and (−) strands, obtained from a single biological experiment.

The following paragraphs summarize some of our best practices in experiment planning and technical validation.

First, the FASTQC software (http://www.bioinformatics.bbsrc.ac.uk/projects/fastqc) was used to quality check the FastQ files obtained from the Illumina Miseq sequencer.

Paired-end reads from the sequencer platform were merged together using the PEAR tool^9^ with a minimum of 40 overlapping residues as the threshold (mean PHREAD score of at least 33). We used very stringent parameters: fragment length threshold, 50%; threshold alignment primers, 80%; bisulfite conversion efficiency, 99% and threshold alignment to reference, 50%. The number of reads selected by the various filters is shown in Table 2. The pipeline output format reports the methylation status for each CpG dinucleotide, coded 0 as non-methylated, 1 if methylated and 2 if the methylation state cannot be assessed. We use this output to perform the analysis.

**Figure 3:**
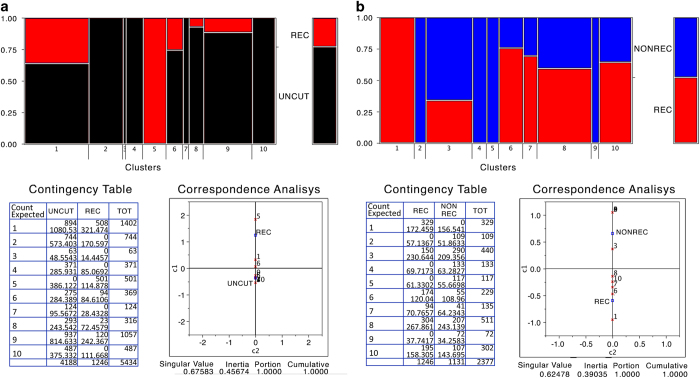
Epiallelic species present in control (UNCUT), recombinant and non- recombinant cells. Cluster analysis of the entire collection of GFP sequences. (**a**) Red and black represent the fraction of molecules present in REC (red) or control UNCUT (black) cells. The panel below on the left represents the actual number of sequences in each cluster. The panel on the right shows the Euclidean distance between the various clusters. (**b**) Red and blue represent the fraction of molecules present in REC (red) or NONREC cells repaired mainly by NHEJ (blue). The panel below on the left represents the actual number of sequences in each cluster. The panel on the right shows the Euclidean distance between the various clusters.

To improve on and validate the analysis of the composition of specific methylated species in control versus non-recombinant or recombinant molecules we performed a clustering analysis of the specific species present in recombinant and non-recombinant groups. Figure 3 shows that there is 1 cluster that is present only in recombinant cells and 4 clusters present only in non-recombinant cells (SAMD00063103, SAMD00063107, Data Citation 1), indicating that the methylation profiles of the HR-repaired gene are not random, but are specific and stable.

To further validate the analysis of methylation, we modified the DNA methylation by manipulating the levels of BER enzymes. One representative BER enzyme, APE1, is involved in DNA demethylation following methyl C oxidation^10^.

**Figure 4:**
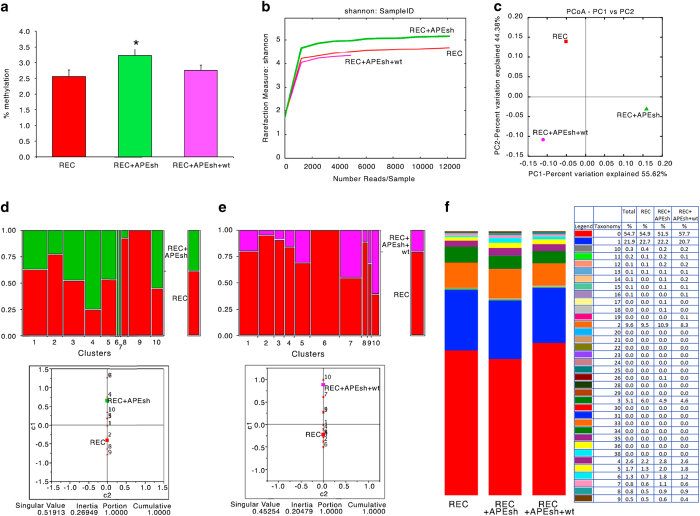
DNA methylation variation induced by manipulating BER (APE1) enzyme levels. (**a**) Quantitative methylation analysis for each of the 41 CpG in GFP gene in REC cells (red) in which APE1 was depleted (green, APEsh) or reconstituted with an expression vector (pink, APEsh+wt). Data were expressed as the mean±s.e.m. (*n*=41); *P<0.01 (paired *t-*test) comparing REC+APEsh versus REC or versus REC+APEsh+wt. (**b**) Shannon diversity index in REC cells (red) in which APE1 was depleted (green, APEsh) or reconstituted with an expression vector (pink, APEsh+wt). (**c**) Principal component analysis in REC cells (red) in which APE1 was depleted (green, APEsh) or reconstituted with an expression vector (pink, APEsh+wt). (**d**) Red and green represent the fraction of molecules present in REC cells (red) or in which APE1 was depleted (green). The panel below shows the Euclidean distance between the various clusters. (**e**) Red and pink represent the fraction of molecules present in REC cells (red) or in which APE1 was reconstituted with an expression vector (pink). The panel below shows the Euclidean distance between the various clusters. (**f**) Methylation content (taxonomy) of in REC cells (red) in which APE1 was depleted (green, APEsh) or reconstituted with an expression vector (pink, APEsh+wt).

**Figure 5:**
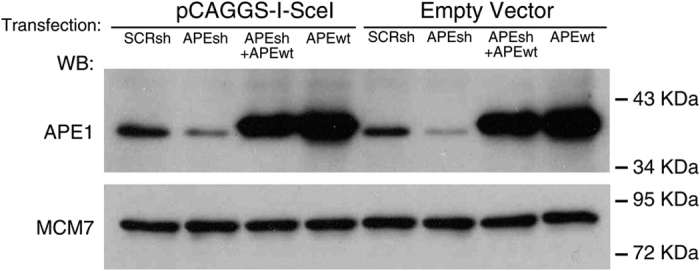
Depletion of BER (APE1) enzyme levels. Immunoblot with specific antibodies to APE1 in cells in which APE1 levels were depleted (APEsh) or reconstituted with an expression vector (APEsh+wt). The cells were transfected or not with I-SceI expression vector (pCAGGS-I-SceI) or scrambled shRNA.

**Figure 6:**
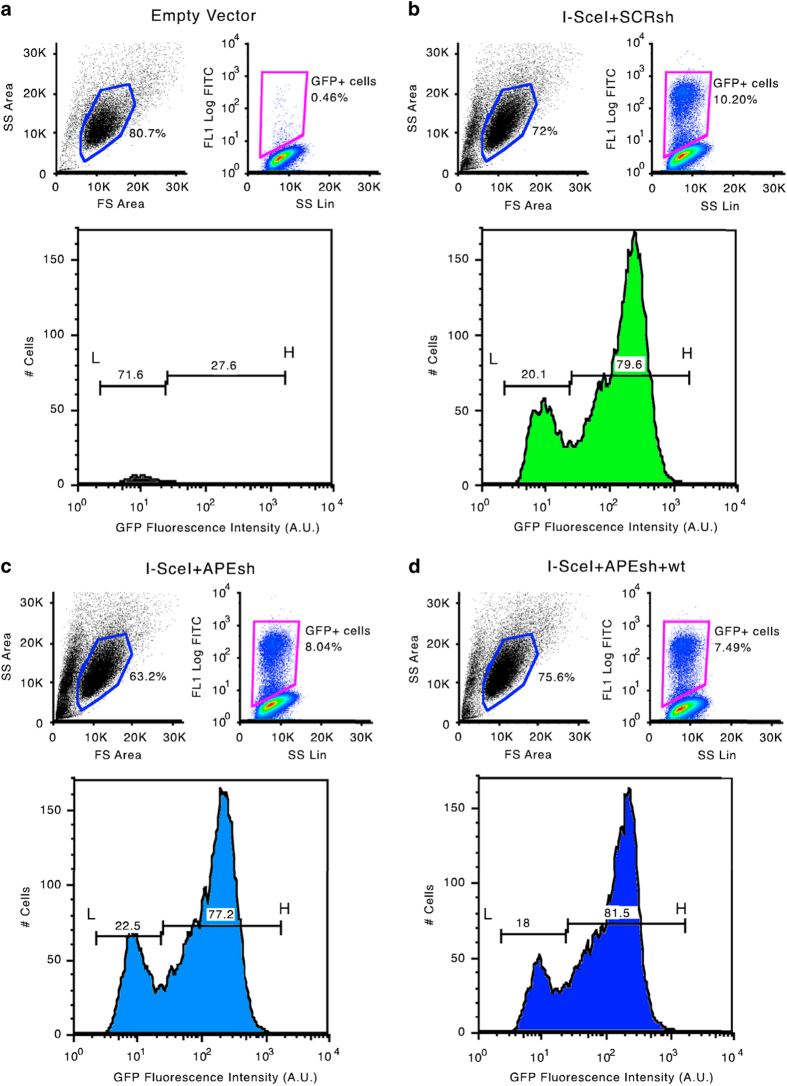
Variation of methylation status and expression of the repaired GFP gene induced by manipulating BER (APE1) levels. Cytofluorimetric analysis of DRGFP cells undergoing repair by HR. (**a**,**b**) show HR GFP cells 7 days after the DSB and HR, including a control without DSB. The upper panels show the gating strategy to visualize GFP positive cells. The lower panels show the distribution of GFP+ cells after HR. The horizontal lines indicate the fraction of low expressers (L) or high expressers (H), which have been extensively characterized^1,3^. (**c**,**d**) Show the gating and the distribution of L and H cells in GFP+ cells, in which APE1 levels were depleted or reconstituted 48 h after DSB.

Figure 4a shows the extent (mean±s.e.m.) of total methylation for each of the 41 CpGs present in the recombinant cassette of GFP molecules in cells in which APE1 was depleted (APEsh) (SAMD00063104, SAMD00063108, Data Citation 1) or reconstituted with an expression vector (APEsh+wt) (SAMD00063105, SAMD00063109, Data Citation 1). There was a significant increase in overall methylation for each CpG, which was suppressed by restoring the wild-type function, consistent with the role of APE1 in C demethylation^10^. Also the similarity index of the methylated molecules present in the three samples was modified by APE1 depletion and partly reconstituted by expressing the wild-type enzyme. This is shown by the similarity of the methylation profiles between recombinant and APE1 reconstituted groups (Fig. 4c, variation 44 versus 55%). Methylation variations are also shown by cytofluorimetric analysis of HR GFP+ cells 7 days after the DSB, in which APE1 levels were depleted or reconstituted (Fig. 5) (WB_APE, Data Citation 2). The HR cells display two fractions of GFP+ cells: on the left of the lower panel b, L cells (20.1%) are shown, which express GFP poorly because the gene is hypermethylated; on the right of lower panel b, H cells (79,6%) are shown; these cells express higher levels of the GFP gene because the gene is hypomethylated^1,3^ (Fig. 6) (bsk 7gg_0, sce scr 7gg_13_Apr_11, Data Citation 3). Panels c and d in Fig. 6 show that APE1 silencing reduces H and increases L cells (sceAPE1 Ref(SH) 7gg_13_Apr_11, Data Citation 3); conversely, the reconstitution of APE1 or Aza dC increases the number and the fluorescence intensity of H and reduce L^1,3,6^ (sce APE(SH)_APE1 wt 7gg_13_Apr_11, Data Citation 3).

Collectively, by using different analytical approaches, we are able to measure HR- specific methylation marks. HR-specific methylation marks are not specific to the cell line we have used (HeLa) but the same patterns were found in mouse ES cells^1,2^. The HeLa cell line used for the experiments described is a pool of clones each containing the reporter gene DRGFP as a single copy insertion. The methylation analysis we describe provides the tools to trace overtime the distribution of various epialleles of any gene, to document their repair history and to quantify the strength of selection for their retention.

## Usage Notes

The DNA-Seq fastq files (n. 8) contain the sequences corresponding to (−) and (+) strands. Recombinants or NON-recombinants or NHEJ molecules were sorted on the basis of the primary DNA sequence. The DNA sequences of NON-recombinant, NHEJ or uncut molecules were converted into recombinant sequence to permit the comparison of molecules differing only by CpG methylation. The sequence of the (−) strand was converted into that of the (+) strand and pooled together with the original (+) strand. The four files analysed correspond to the (+) strand.

To analyse the methylation status of each amplicon, we used the AMPLIMETHPROFILER^11^ specifically designed for deep-targeted bisulfite amplicon sequencing of multiple genomic regions. This pipeline is freely available at https://sourceforge.net/projects/amplimethprofiler and is organized as follows: first, it recognizes corresponding target regions discarding PCR artefacts and reads that do not match expected lengths; then, reads are aligned to the corresponding bisulfite- converted reference using BLASTn^12^. This output is subjected to the analysis. The quantitative methylation average for each site is represented by the ratio between the number of non-converted bases at that site and the total number of mapped reads. The abundance of each of the 2^NCpG^ distinct epialleles (where NCpG denotes the number of CpG sites in the region analysed) was evaluated for each sample by counting the number of passing filter reads containing that epiallele. Qualitative methylation analysis was performed using Qiime^13^, which includes: (1a)*’summary’* the number of profiles present in each input sample; (2a) *‘taxa_summary_plots’* information on the distribution of methylation profile classes; (3) ‘*alpha diversity’* the five alpha diversity metrics for each sample: a) the number of different methylation profiles in the sample b) the Shannon entropy c) the Simpson index d) the Chao 1 index e) number of singletons (such metrics were computed through a rarefaction procedure to take into account biases derived from variable sequencing depth) and; (4) ‘beta diversity’,i.e., the distance between samples in terms of the composition of their methylation profiles, measured by Bray-Curtis dissimilarity: (5) pPrincipal coordinates analysis (PCoA).

## Additional Information

**How to cite this article:** Pezone, A. *et al.* High-coverage methylation data of a gene model before and after DNA damage and homologous repair. *Sci. Data* 4:170043 doi: 10.1038/sdata.2017.43 (2017).

**Publisher’s note:** Springer Nature remains neutral with regard to jurisdictional claims in published maps and institutional affiliations.

## Supplementary Material



## Figures and Tables

**Table 1:** List of DNA oligonucleotides used for PCR.

**ID**	**PRIMERS**	**Locus**
Minus/Bisulfite E3F	5′- GTATTTTAGTTTGTGTTTTAGGATG-3′	pDR-GFP
Minus/Bisulfite E4R	5′- CACCTAAAACTAAAACACT-3′	pDR-GFP
Plus/Bisulfite E5F	5′- AGGAGGTATTTGGAGTTGAGGTA-3′	pDR-GFP
Plus/Bisulfite E6R	5′- TACTCCAACTTATACCCCAAAATAT-3′	pDR-GFP

**Table 2:** Samples quality and reads statistics.

**Source**	**Transfection**	**Bisulfite conversion efficiency**	**Total Reads ×10**^**6**^ **(before filtering)**	**Total reads ×10**^**6**^ **(after filtering)**	**Reads with I-Sce I site**	**Reads with Bcg I site**	**% of I-Sce I site**	**% of Bcg I site**
HELA_DRGFP (minus_strand)	BSK	99%	85.86	81.92	42.41	39.5	49.39	46.01
HELA_DRGFP (minus_strand)	SCE	99%	29.66	29	12.05	16.94	40.64	57.12
HELA_DRGFP (minus_strand)	SCE +APEsh	99%	11.18	10.92	3.2	7.72	28.66	69.03
HELA_DRGFP (minus_strand)	SCE +APEsh +APEwt	99%	5.63	5.5	1.84	3.65	32.73	64.95
HELA_DRGFP (plus_strand)	BSK	99%	56.86	54.21	26.03	28.17	45.78	49.54
HELA_DRGFP (plus_strand)	SCE	99%	14.08	13.64	5.07	8.56	36.02	60.81
HELA_DRGFP (plus_strand)	SCE +APEsh	99%	7.55	7.26	1.66	5.6	21.99	74.2
HELA_DRGFP (plus_strand)	SCE +APEsh +APEwt	99%	2.46	2.38	577	1.81	23.4	73.5
